# Trends in the Main Residency Match From 2007 to 2020

**DOI:** 10.7759/cureus.53968

**Published:** 2024-02-10

**Authors:** Benjamin W Van, Micaela White, Om Patel, Dagoberto Pina, Joseph B Wick, Hai V Le

**Affiliations:** 1 Orthopaedics, UC Davis Health, Sacramento, USA

**Keywords:** abstract presentation publication, match trends, step 1 change, research, usmle step 1 pass/fail

## Abstract

Background

The United States Medical Licensing Exam (USMLE) Step 1 was recently changed from a numerically scored grading system to a pass/fail grading system. Until late 2024, there will be no formal studies about the impact that the grading change will have on the match process. To thoroughly assess the impact that this change will have on the overall match process, it is important to look at what the trends in applicants’ objective measures have been in the years before the change. We aim to systematically evaluate the rates of change and mean trend of objective metrics found in residency applications in the main residency match.

Methods

Objective medical student data of matched and unmatched applicants were queried from the National Matching Program’s Charting Outcomes in the Match Reports for the 2007 to 2020 application cycles. Data were used to create linear regression analyses and statistical tests were performed to evaluate trends over time.

Results

For matched applicants, there were statistically significant positive trends for the mean number of contiguous ranks (m=0.33, p<0.01), having another non-doctoral graduate degree (m=0.67, p<0.01), membership to Alpha Omega Alpha (AOA) honor society (m=0.22, p<0.01), mean USMLE Step 1 score (m=1.01, p<0.01), mean USMLE Step 2 score (m=1.68, p<0.01), mean number of research experiences (m=0.12, p<0.01), and mean number of abstracts, presentations, and publications (m=0.34, p<0.01). Additionally, there was a statistically significant negative trend for the percentage who graduated from a top 40 National Institutes of Health-funded medical school (m=-0.41, p<0.01). For unmatched applicants, there were statistically significant positive trends for having another non-doctoral graduate degree (m=0.83, p<0.01), mean USMLE Step 1 score (m=1.26, p<0.01), mean USMLE Step 2 score (m=2.27, p<0.01), mean number of research experiences (m=0.13, p<0.01), and mean number of abstracts, presentations, and publications (m=0.33, p<0.01).

Conclusion

Our study shows that there have been statistically significant increases in almost all objective measures in the residency application. Recent changes to the abstracts, presentations, and publications on the Step 1 scoring system will force almost all residency programs to overhaul their application process and potentially increase reliance on Step 2, research, and other nonobjective factors. For students early in their medical education, emphasis on Step 2 and research will yield increased chances of matching into residency in the future.

## Introduction

Annually, thousands of senior medical students apply for residency programs through an extensive process known as the Match. The Match, hosted by the National Residency Medical Program (NRMP), compiles applications and uses an algorithm that pairs applicants with residency programs based on mutual preferences [1]. Matching into a residency program is a crucial component of becoming a practicing physician in the United States, although there are many senior medical students every year who do not match [2]. Critical to matching is having a strong application, and while many factors are used to determine how competitive a prospective applicant is, some objective measurements that are commonly used are the United States Medical Licensing Exam (USMLE) Step 1 and Step 2 scores, research productivity, attendance to a top 40 National Institutes of Health (NIH)-funded medical school, and membership into honor societies [3-5].

Out of these objective measurements, USMLE Step 1 has garnered attention recently as the National Board of Medical Examiners (NBME) moved to change its numerical scoring into a solely pass/fail scoring system in early 2022 [6]. Proponents of the change state that the USMLE Step 1 score had a disproportionate importance in the residency match process and a negative impact on student well-being and the medical school curriculum [7]. Those who oppose the change state that the lack of this objective score will make it harder for residency programs to compare candidates from different schools. Therefore, students from less preeminent medical schools may be offered fewer interviews or experience a lower likelihood of matching [8]. Currently, there are no formal studies regarding the impact of the Step 1 grading change on the match process because the first medical school graduating class with the grading change is not expected until 2024.

To thoroughly assess the impact that the Step 1 grading change will have on the overall match process, it is important to examine the trends in the applicant’s objective measures in the years before the change. Therefore, in this study, we assessed trends in the NRMP main residency application data spanning from 2007 to 2020. The evaluation encompassed factors such as USMLE scores, research productivity, top 40 NIH-funded medical school attendance, additional degrees, and Alpha Omega Alpha (AOA) status. Information on trends in factors other than USMLE Step 1 scores may be useful for medical students building their applications and medical student advisors because early data on residency match success in the post-USMLE Step 1 scoring era will take several years to accrue.

## Materials and methods

Study design and setting

This was a retrospective survey of a large, longitudinally maintained database retrieved from the National Resident Matching Program’s Charting Outcomes in the Match Reports for the 2007 to 2020 application cycles [9]. These National Resident Matching Program’s reports are published biennially and are available online to the public. We believe this report is ultimately the most comprehensive and up-to-date report for the main residency match applicant objective metrics.

Participants

All medical students attending a Liaison Committee on Medical Education-accredited allopathic medical school were included in this study, which is a total of 111,983 applicants. Data were obtained to evaluate trends in characteristics of senior medical students from allopathic medical schools applying in the Main Residency Match from 2007 to 2020. Therefore, students from osteopathic medical schools and international medical graduates (IMGs) were not included in the study.

Data extracted comprised several matched and unmatched applicants, number of contiguous ranks (defined as the number of programs any individual applicant ranked within one specialty before listing a different specialty), USMLE Step 1 and Step 2 scores, number of abstracts/presentations/publications (A/P/P), Top 40 NIH medical school status, additional degrees, and AOA status.

Study outcomes 

The primary goal of our study was to assess the trends of objective factors to determine the one that had the greatest rate of change during the study period. To achieve this, we calculated rates of change for each objective metric obtained.

Ethical approval 

Per the policy of our institution, institutional review board approval is not required for data that are publicly available and de-identified. Thus, ethical approval for this study was not sought.

Statistical analysis 

National Resident Matching Program’s Charting Outcomes in the Match Reports data contain the information of all allopathic medical student applicants. Therefore, no sampling was necessary, and confounding factors were not considered. The data fit all criteria to perform a simple linear regression - linearity, independence of errors, normality of errors, and equal variances. Each objective metric as listed above except for the number of matched and unmatched applicants was divided into a matched applicant and unmatched applicant category, and linear regression analyses were subsequently performed to evaluate trends over time (from 2007 to 2020). For statistical testing, our null hypothesis was no change in the objective metric from 2007 to 2020 (slope = 0), while our alternative hypothesis was an increase or decrease in the objective metric from 2007 to 2020 (slope ≠ 0). P values were calculated to determine if we could reject our null hypothesis. The level of significance was set at p = 0.05. Statistical tests were performed using Stata version 17 (StataCorp, College Station, TX).

## Results

Number of matched and unmatched applicants

In 2007, there were 14,420 allopathic applicants in the main residency match and, in 2020, there were 17,585 applicants. The mean number of matched allopathic applicants was 14,614, and the mean number of unmatched allopathic applicants was 1,383. The number of applicants in the main residency match has grown by about 243 applicants per year, with a brief decline in 2016. Furthermore, in 2007, there were 1,157 (8.0%) unmatched allopathic applicants in the main residency match, and, in 2020, there were 1,527 (7.7%) unmatched applicants. The number of unmatched applicants has grown by about 28 (~2.0%) per year with a brief spike in unmatched applicants in 2016.

Overall, the match rate was 92.0% in 2007 and 91.3% in 2020. From 2007 to 2020, the mean match percentage for allopathic applicants was 91.4% with a brief dip in 2016 when the match rate was 89.1% (Table 1).

**Table 1:** Number of Matched Applicants, Unmatched Applicants, and Overall Match Rate in the Main Residency Match from 2007 to 2020

	2007	2009	2011	2014	2016	2018	2020
MD Matched	13,263	13,646	14,237	15,127	14,518	15,451	16,058
MD Not Matched	1,157	1,312	1,340	1,245	1,766	1,336	1,527
Match Rate	92.0%	91.2%	91.4%	92.4%	89.2%	92.0%	91.3%

Contiguous ranks

**Figure 1:**
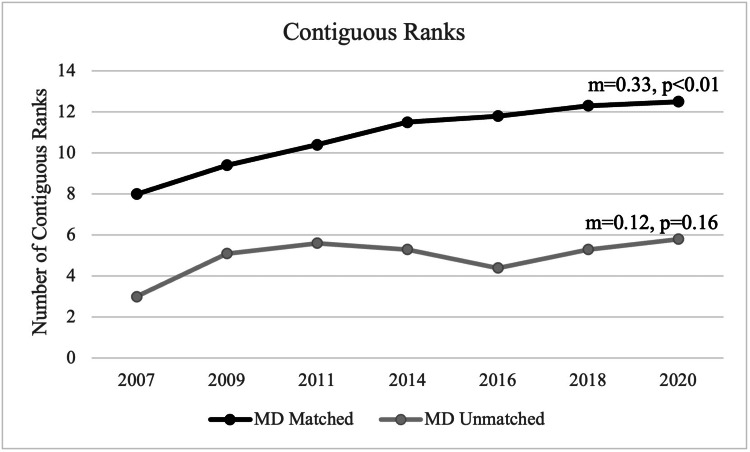
Contiguous Ranks Between MD Matched and Unmatched Applicants

From 2007 to 2020, the number of contiguous ranks for applicants that have matched increased from 8 to 12.5 with a statistically significant positive trend (m=0.33, p<0.01) and a mean of 10.8 contiguous ranks during this period. For unmatched applicants in the main residency match, the mean number of contiguous ranks was 4.9. Although there was a notable increase in the number of programs ranked between 2007 and 2009, there was no statistically significant trend observed in the number of contiguous ranks for unmatched applicants (m=0.12, p=0.16) (Figure 1).

Number of specialties ranked

**Figure 2:**
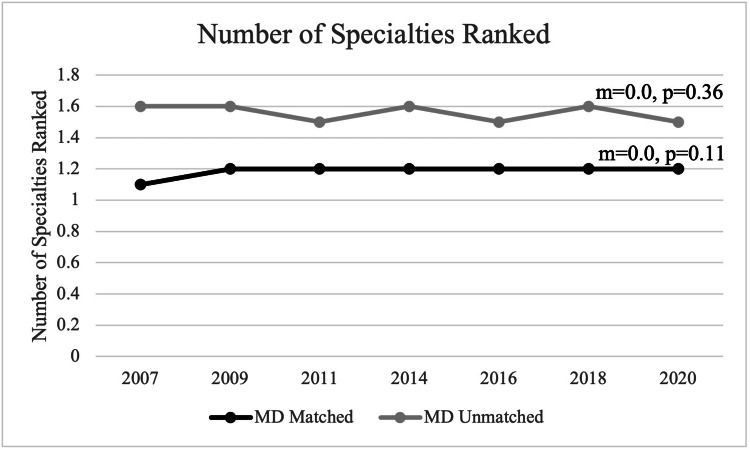
Number of Specialties Ranked Between MD Matched and Unmatched Applicants

Among the matched allopathic applicants overall, slightly more applicants began applying to more than one specialty between 2007 and 2009 with the mean number of specialties ranked increasing from 1.1 to 1.2 between 2007 and 2009 and remaining constant with no statistically significant trend observed (m=0.0, p=0.11). Among unmatched allopathic applicants between 2007 and 2020, they applied to a mean of 1.6 different specialties with no statistically significant trend observed as well (m=0.0, p=0.36) (Figure 2).

Medical school rank in NIH funding

**Figure 3:**
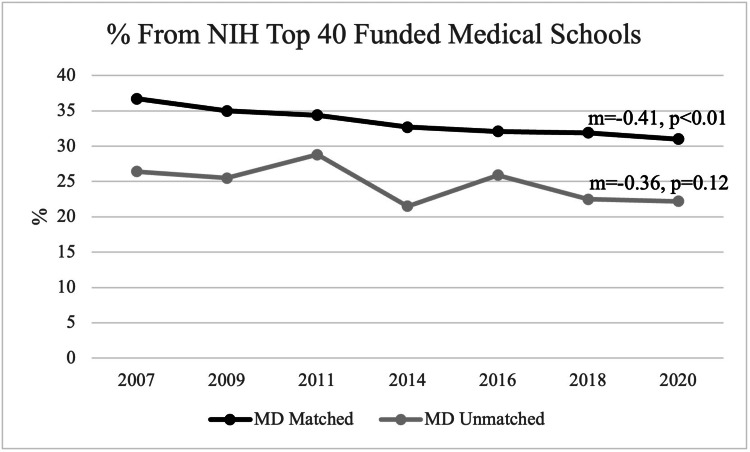
Percentage of Students from Top 40 NIH-Funded Schools Between Matched and Unmatched Applicants

For matched applicants, there was a mean of 33.4% of applicants who attended a Top 40 NIH-funded medical school during the observed period. In 2007, among all matched applicants, 36.7% (4,907) attended a Top 40 NIH-funded medical school, while in 2020, 31.0% (4,978) of matched applicants attended a Top 40 NIH-funded medical school. Overall, there has been a statistically significant decline in the percentage of matched allopathic applicants from the Top 40 NIH-funded medical schools since 2007 (m=-0.41, p<0.01). Among unmatched applicants, on average, about 24.7% are from the Top 40 NIH-funded medical schools. However, there was no statistically significant trend appreciated over this period (m=-0.36, p=0.12) (Figure 3).

Additional graduate degree

**Figure 4:**
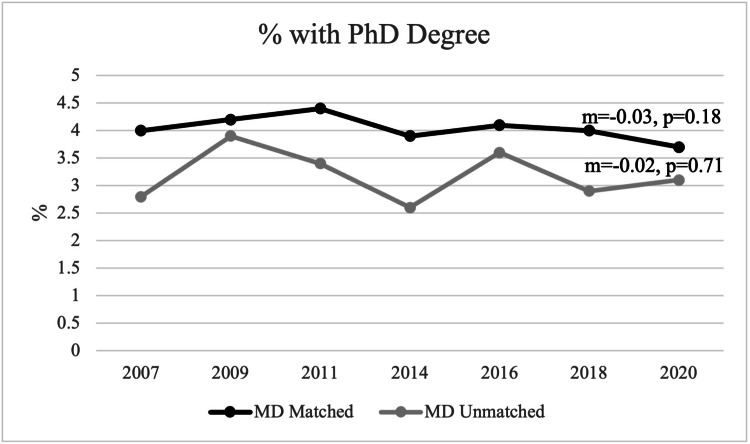
Percentage of Students with an Additional PhD Degree Between Matched and Unmatched Applicants

From 2007 to 2020, less than 5% of applicants have a PhD for both matched and unmatched applicants. For matched applicants, there was a mean of 4.0% of applicants having a PhD degree, and, for unmatched applicants, there was a mean of 3.2% of applicants having a PhD degree. There was no statistically significant trend in applicants who also had a PhD degree from 2007 to 2020 for both matched and unmatched applicants (matched: m=-0.03, p=0.18; unmatched: m=-0.02, p=0.71) (Figure 4).

**Figure 5:**
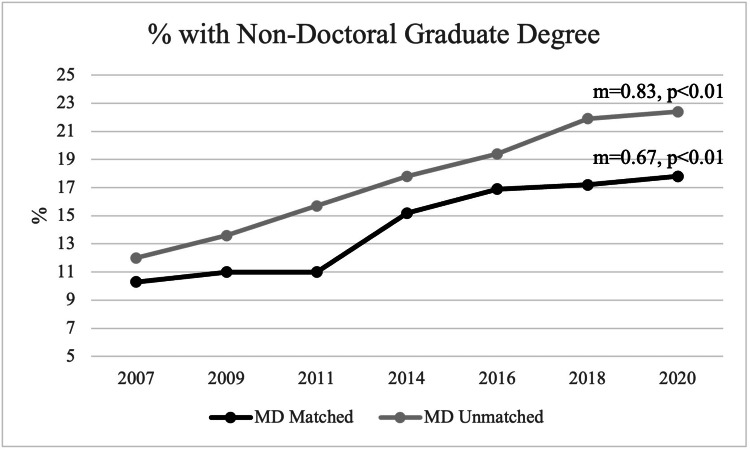
Percentage of Students with an Additional Non-Doctoral Graduate Degree Between Matched and Unmatched Applicants

In contrast, the percentage of allopathic applicants obtaining a non-doctoral graduate degree has increased in both matched and unmatched applicants. In 2007, about 10.3% (1,366) of matched applicants had an additional non-doctoral graduate degree, and, in 2020, about 17.8% (2,858) of matched applicants had an additional non-doctoral graduate degree with a statistically significant positive trend noted over time (m=0.67, p<0.01). For unmatched applicants, in 2007, 12% (139) had an additional non-doctoral graduate degree, and, in 2020, 22.4% (342) had an additional non-doctoral graduate degree. A statistically significant positive trend is also observed in unmatched applicants regarding non-doctoral graduate degrees (m=0.83, p<0.01) (Figure 5).

AOA membership

**Figure 6:**
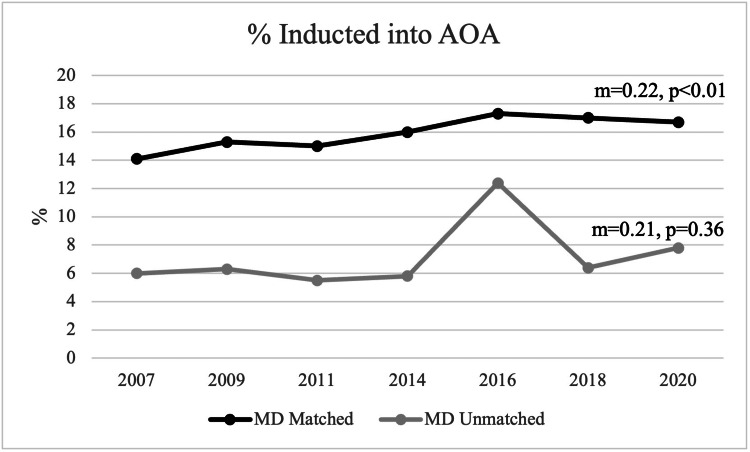
Percentage of Students Inducted into AOA Between Matched and Unmatched Applicants

For matched applicants, there was a mean of 15.9% being members of AOA during the observed period. In 2007, 14.1% (1,870) of matched applicants were members of AOA, and, in 2020, 16.7% (2,682) of matched applicants were members of AOA. A statistically significant positive trend was noted in the percentage of AOA membership for matched applicants (m=0.22, p<0.01). For unmatched applicants, there was a mean of 7.2% being members of AOA. However, there was no statistically significant trend observed (m=0.21, p=0.36) (Figure 6).

USMLE Step 1 and Step 2 exam scores

**Figure 7:**
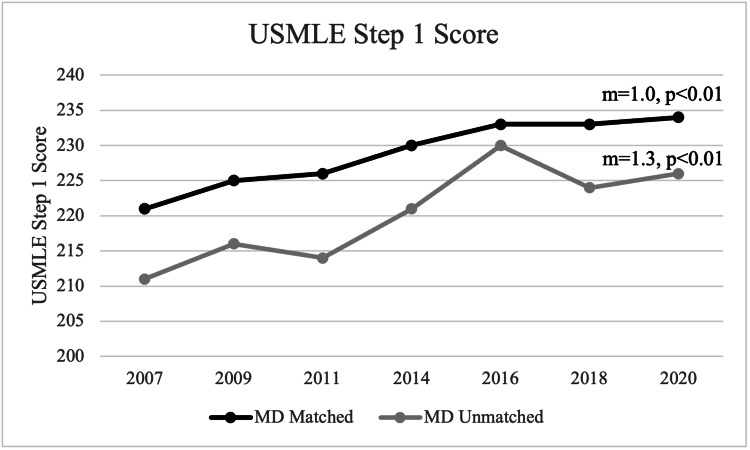
USMLE Step 1 Score Between Matched and Unmatched Applicants

For matched applicants, the mean USMLE Step 1 score was 229 during the observed period. In 2007, the mean USMLE Step 1 score was 221, and, in 2020, the mean USMLE Step 1 score was 234 for matched applicants. For unmatched applicants, the mean USMLE Step 1 score was 220 during the observed period. In 2007, the mean USMLE Step 1 score was 211, and, in 2020, the mean USMLE Step 1 score was 226 for unmatched applicants. A statistically significant positive trend was noted for the USMLE Step 1 score for both matched and unmatched applicants (matched: m=1.0, p<0.01; unmatched: m=1.3, p<0.01) (Figure 7).

**Figure 8:**
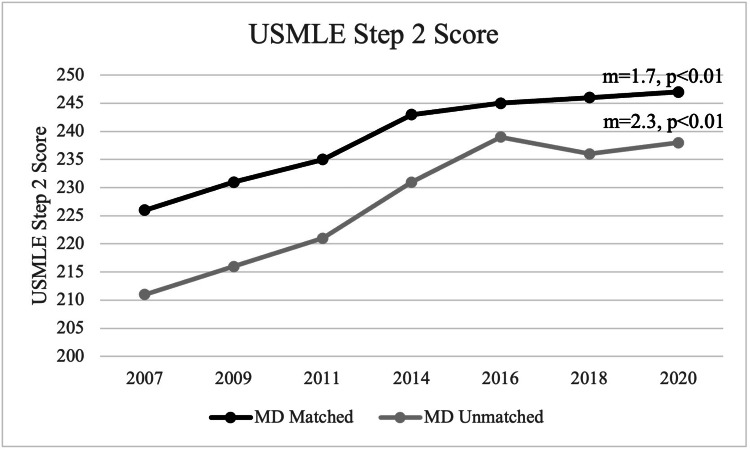
USMLE Step 2 Score Between Matched and Unmatched Applicants

For matched applicants, the mean USMLE Step 2 score was 239 during the observed period. In 2007, the mean USMLE Step 2 score was 226, and, in 2020, the mean USMLE Step 2 score was 247 for matched applicants. For unmatched applicants, the mean USMLE Step 2 score was 227 during the observed period. In 2007, the mean USMLE Step 2 score was 211, and, in 2020, the mean USMLE Step 2 score was 238 for unmatched applicants. A statistically significant positive trend was noted for the USMLE Step 2 score for both matched and unmatched applicants (matched: m=1.7, p<0.01; unmatched: m=2.3, p<0.01) (Figure 8).

Research experiences and abstracts/presentations/publications

**Figure 9:**
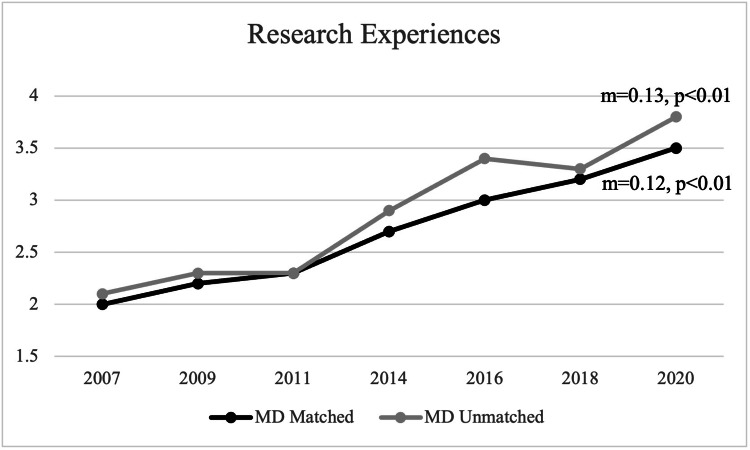
Number of Research Experiences Between Matched and Unmatched Applicants

For matched applicants, the mean number of research experiences was 2.7 during the observed period. In 2007, the mean number of research experiences was two, and, in 2020, the mean number of research experiences was 3.5 for matched applicants. For unmatched applicants, the mean number of research experiences was 2.9 during the observed period. In 2007, the mean number of research experiences was 2.1, and, in 2020, the mean number of research experiences was 3.8 for unmatched applicants. A statistically significant positive trend was noted for the number of research experiences for both matched and unmatched applicants (matched: m=0.12, p<0.01; unmatched: m=0.13, p<0.01) (Figure 9).

**Figure 10:**
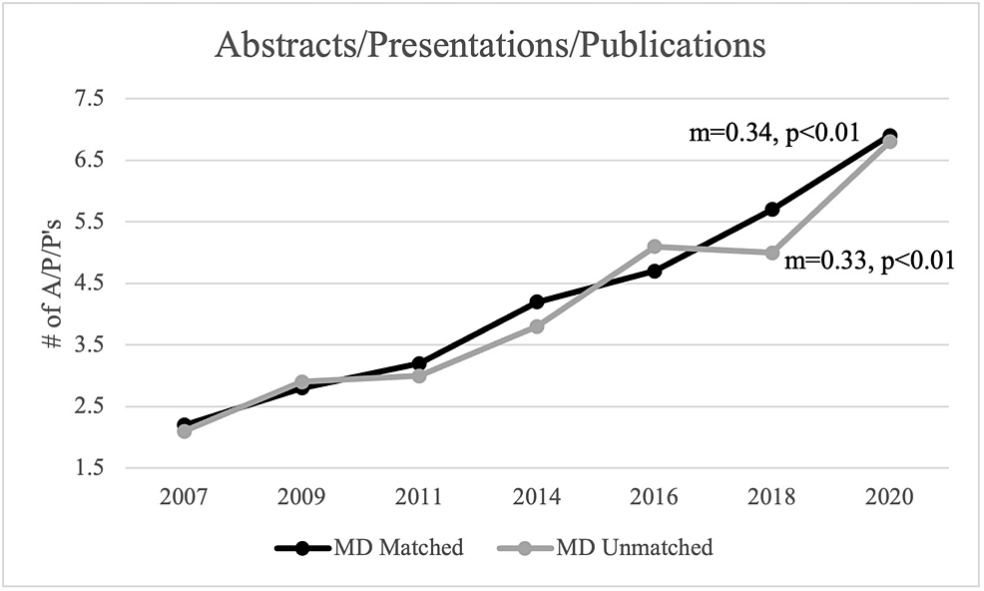
Number of A/P/Ps Between Matched and Unmatched Applicants

For matched applicants, the mean number of A/P/Ps was 4.2 during the observed period. In 2007, the mean number of A/P/Ps was 2.2, and, in 2020, the mean number of A/P/Ps was 6.9 for matched applicants. For unmatched applicants, the mean number of A/P/Ps was 4.1 during the observed period. In 2007, the mean number of A/P/Ps was 2.1, and, in 2020, the mean number of A/P/Ps was 6.8. A statistically significant positive trend was noted for the number of A/P/Ps for both matched and unmatched applicants (matched: m=0.34, p<0.01; unmatched: m=0.33, p<0.01) (Figure 10).

## Discussion

Our study aims to evaluate trends in the NRMP from 2007 to 2020 to identify any objective measures that may have significantly impacted applicants’ match process before Step 1 moved to a pass/fail scoring system in 2022. Through our evaluation of the NRMP data, our study finds that even though the match rate has been consistent between 2007 and 2020, there have been statistically significant trends in some objective measures used to evaluate applicants for the residency match. Our analysis found statistically significant positive trends for matched applicants in the mean number of contiguous ranks, having another non-doctoral graduate degree, membership to AOA, mean USMLE Step 1 score, mean USMLE Step 2 score, mean number of research experiences, and mean number of A/P/Ps. Additionally, there was a statistically significant negative trend for the percentage of those who graduated from a top 40 NIH-funded medical school. For unmatched applicants, there were statistically significant positive trends for having another non-doctoral graduate degree, mean USMLE Step 1 score, mean USMLE Step 2 score, mean number of research experiences, and mean number of A/P/Ps.

The more interviews and thus the more contiguous ranks that an applicant has on their final rank list, the more likely they are to match. Our data showed that matched applicants had a statistically significant increase in contiguous ranks from 2007 to 2020, while unmatched applicants did not. As applicants can only rank a program after they have been interviewed, matched applicants were therefore likely to have completed more interviews than unmatched applicants. However, it is uncertain whether the increased number of interviews is because of applicants recently applying to more programs than before [10]. Regardless, it appears that applicants can accurately perceive the strength of their applications. For example, those who successfully matched consistently applied to 1.2 specialties, whereas unmatched applicants tended to apply to 1.6 specialties meaning that they applied to an additional specialty as a “backup.” Additionally, applicants who did not match were found to have additional non-doctoral graduate degrees. However, it is uncertain what the impact of the additional degree has on their application. 

Another factor that saw statistically significant positive trends in applicants was USMLE Step 1 and Step 2 scores. Historically, higher board scores have been favored by programs as they provide an objective measure of applicants, and this is further evidenced by several studies as a proxy for resident clinical performance and performance on in-training exams [11-12]. While cutoffs for USMLE board scores may correlate with potential residency success, they may also diminish equity among applicants and reduce diversity among residency programs and specialties [13]. Some authors believe that USMLE cutoffs are in place because of an overwhelming number of applications per residency seat and that a radical reform of the residency application process should be conducted so that applicants can be evaluated more holistically [14]. Some proposed solutions are putting a cap on the number of programs an applicant can apply to [10] or having applicants designate some programs as “preferred programs” [15].

There were statistically significant increases in the number of research experiences and A/P/Ps for both matched and unmatched applicants in the main residency match. Surprisingly, in the most recent year analyzed, 2020, unmatched applicants had, on average, more research experiences and almost the same number of A/P/Ps as matched applicants. One possible explanation for this is in line with what was previously mentioned where applicants accurately perceive the strength of their applications and compensate for possible lower USMLE Step 1 and Step 2 scores through research experiences and productivity [16-17]. Now that USMLE Step 1 has become pass/fail, many residency program directors, especially those in competitive fields such as orthopedic surgery agree that research experiences will become more important [16-19]. A study completed in 2017 evaluated trends in the NRMP data between 2006 and 2014 for the orthopedic match only. They found that the mean number of research works was significantly higher for those who matched versus those who did not [20]. While this study was done before Step 1 moved to a pass/fail model, it still supports a need for high research productivity for competitive specialties, such as orthopedics. This further emphasizes our hypothesis that the mean number of abstracts, posters, and presentations will become increasingly important with the changed Step 1 scoring model. A study completed in 2021 determined that 40% of residency interviewers asked applicants about their research projects during the 2019-2020 application cycle. This study also found that interviewers at academic institutions asked about research experiences at a statistically significant increased rate compared to interviewers at nonacademic programs [21]. Although this study was completed at a single institution, this helps justify the importance of research experiences for medical students when applying to residency programs, highlighting its prevalence in the interview evaluation process. Additionally, it provides valuable information for applicants who are interested in attending a residency program at an academic institution versus community-based programs.

Our study is limited by its retrospective nature and the utilization of the NRMP summary data, limiting our ability to perform direct statistical comparisons between applicants. This study solely looked at the main residency match as a whole and did not account for potential changes in individual specialties. Additional factors, both academic and nonacademic, and the emphasis programs place on them were not accounted for. Recent changes in USMLE scoring and changes related to the COVID-19 pandemic are not wholly accounted for in the 2007 to 2020 NRMP data. Despite these limitations, our study is the first to collate long-term, objective NRMP data on all allopathic applicants from 2007 to 2020 and evaluate its trends.

## Conclusions

In conclusion, our study shows that there have been statistically significant increases in almost all objective measures in the residency application. Recent changes to the USMLE Step 1 scoring system to pass/fail will force almost all residency programs to overhaul their application process and potentially increase reliance on Step 2, research, and other nonobjective factors. Our study agrees with this analysis as we found that before the scoring change, there already were statistically significant increases in Step 2 scores and research productivity. More emphasis will be placed on these other aspects with the greatest being on “quantitative” measures, such as Step 2 score and research experiences and productivity. For students early in their medical education, we believe that, for the time being, before more studies can be done, putting emphasis on Step 2 and research will yield increased chances of matching into residency in the future.

## References

[1] (2022). Your guide to the main residency match. https://www.nrmp.org/residency-applicants/.

[2] (2020). Medical school enrollments grow, but residency slots haven’t kept pace. https://www.aamc.org/news/medical-school-enrollments-grow-residency-slots-haven-t-kept-pace.

[3] Gauer JL, Jackson JB (2017). The association of USMLE Step 1 and Step 2 CK scores with residency match specialty and location. Med Educ Online.

[4] Matthews CN, Estrada DC, George-Weinstein M, Claeson KM, Roberts MB (2019). Evaluating the influence of research on match success for osteopathic and allopathic applicants to residency programs. J Am Osteopath Assoc.

[5] Rinard JR, Mahabir RC, Shenoy AB, Mahabir RC (2010). Successfully matching into surgical specialties: an analysis of national resident matching program data. J Grad Med Educ.

[6] (2022). USMLE Step 1 transition to pass/fail only score reporting. https://www.usmle.org/usmle-step-1-transition-passfail-only-score-reporting.

[7] Lin GL, Nwora C, Warton L (2020). Pass/fail score reporting for USMLE Step 1: an opportunity to redefine the transition to residency together. Acad Med.

[8] Dougherty PJ (2021). Corr® curriculum-orthopaedic education: changing USMLE Step 1 scores to pass/fail removes an objective measure of medical knowledge. Clin Orthop Relat Res.

[9] (2022). Main residency match data and reports. Accessed.

[10] Weissbart SJ, Kim SJ, Feinn RS, Stock JA (2015). Relationship between the number of residency applications and the yearly match rate: time to start thinking about an application limit?. J Grad Med Educ.

[11] Panda N, Bahdila D, Abdullah A, Ghosh AJ, Lee SY, Feldman WB (2021). Association between USMLE Step 1 scores and in-training examination performance: a meta-analysis. Acad Med.

[12] Sharma A, Schauer DP, Kelleher M, Kinnear B, Sall D, Warm E (2019). USMLE Step 2 CK: best predictor of multimodal performance in an internal medicine residency. J Grad Med Educ.

[13] Edmond MB, Deschenes JL, Eckler M, Wenzel RP (2001). Racial bias in using USMLE step 1 scores to grant internal medicine residency interviews. Acad Med.

[14] Pereira AG, Williams CM, Angus SV (2019). Disruptive innovation and the residency match: the time is now. J Grad Med Educ.

[15] Whipple ME, Law AB, Bly RA (2019). A computer simulation model to analyze the application process for competitive residency programs. J Grad Med Educ.

[16] Cotter EJ, Polce EM, Williams KL, Spiker AM, Grogan BF, Lang GJ (2022). Current state of research gap-years in orthopedic surgery residency applicants: program directors’ perspectives. Iowa Orthop J.

[17] Cotter EJ, Polce EM, Lee E, Williams KL, Spiker AM, Grogan BF, Lang GJ (2021). Incidence of research gap years in orthopaedic residency applicants: the new standard?. J Am Acad Orthop Surg Glob Res Rev.

[18] Moon J, Khan S, Tao S, Kim G (2023). Analysis of reapplicants to ophthalmology residency: factors associated with successful matching. Semin Ophthalmol.

[19] Yu N, Hoch JS, Martin AR, Shahlaie K (2023). Trends in successfully matched neurosurgery residency applicants. J Neurosurg.

[20] Schrock JB, Kraeutler MJ, Dayton MR, McCarty EC (2017). A comparison of matched and unmatched orthopaedic surgery residency applicants from 2006 to 2014: data from the National Resident Matching Program. J Bone Joint Surg Am.

[21] Daus K, McEchron M (2021). The impact of medical student research as a discussion topic during the residency interview process. BMC Med Educ.

